# Engaging European society at the forefront of cancer research and care: How discussions at the 5^th^ Gago Conference on European Science policy led to the Heidelberg Manifesto

**DOI:** 10.1002/1878-0261.13423

**Published:** 2023-05-05

**Authors:** Michael Baumann, Julio Celis, Ulrik Ringborg, Manuel Heitor, Anton Berns, Tit Albreht, Jeliazko Arabadjiev, Michael Boutros, Mario Brandenburg, Helena Canhao, Fatima Carneiro, Christine Chomienne, Francesco De Lorenzo, Alexander M. M. Eggermont, Angel Font, Elena Garralda, Margarida Goulart, Rui Henrique, Mark Lawler, Lena Maier‐Hein, Francoise Meunier, Simon Oberst, Pedro Oliveira, Maria Papatriantafyllou, Joachim Schüz, Eric Solary, Alfonso Valencia, Rosalia Vargas, Elisabete Weiderpass, Nils Wilking

**Affiliations:** ^1^ German Cancer Research Center (DKFZ) Heidelberg Germany; ^2^ European Academy of Cancer Sciences Stockholm Sweden; ^3^ Danish Cancer Society Research Center Copenhagen Denmark; ^4^ Cancer Center Karolinska Karolinska University Hospital Stockholm Sweden; ^5^ Center for Innovation, Technology and Policy Research, IN+ @ IS Técnico University of Lisbon Portugal; ^6^ The Netherlands Cancer Institute Amsterdam The Netherlands; ^7^ National Institute of Public Health of Slovenia Ljubljana Slovenia; ^8^ Faculty of Medicine University of Ljubljana Slovenia; ^9^ Clinic of Medical Oncology University Hospital Acibadem City Clinic Tokuda Sofia Bulgaria; ^10^ Bulgarian Scientific Society of Immuno‐oncology, and MoC Board Sofia Bulgaria; ^11^ Division Signaling and Functional Genomics German Cancer Research Center (DKFZ) and Heidelberg University Germany; ^12^ DKFZ‐Hector Cancer Institute at the University Medical Center Mannheim Germany; ^13^ Federal Ministry of Education and Research (BMBF) Berlin Germany; ^14^ Comprehensive Health Research Center (CHRC), NOVA Medical School Universidade Nova de Lisboa Portugal; ^15^ Instituto de Patologia e Imunologia Molecular da Universidade do Porto (Ipatimup) Portugal; ^16^ Instituto de Investigação e Inovação em Saúde (i3S), Faculdade de Medicina da Universidade do Porto (FMUP) Portugal; ^17^ Centro Hospitalar Universitário de São João (CHUSJ) Porto Portugal; ^18^ Université de Paris France; ^19^ European Cancer Patient Coalition Brussels Belgium; ^20^ Department Cancer Medicine CSO Princess Máxima Centre Pediatric Oncology, University Medical Center Utrecht The Netherlands; ^21^ Board of the Comprehensive Cancer Center Munich Technical University Munich Germany; ^22^ Ludwig Maximiliaan University Munich Germany; ^23^ La Caixa Foundation Barcelona Spain; ^24^ Vall d'Hebron University Hospital, Vall d'Hebron Institute of Oncology (VHIO) Barcelona Spain; ^25^ Cancer Core Europe Amsterdam The Netherlands; ^26^ Joint Research Center, European Commission Brussels Belgium; ^27^ Department of Pathology & Cancer Biology & Epigenetics Group – Research Center of IPO Porto (CI‐IPOP)/RISE@CI‐IPOP (Health Research Network) Portuguese Oncology Institute of Porto (IPO‐Porto)/Porto Comprehensive Cancer Centre Raquel Seruca (P.CCC Raquel Seruca) Portugal; ^28^ Department of Pathology and Molecular Immunology, School of Medicine & Biomedical Sciences University of Porto (ICBAS‐UP) Portugal; ^29^ FRCPath Patrick G Johnston Centre for Cancer Research, Faculty of Medicine, Health and Life Sciences Queen's University Belfast UK; ^30^ Intelligent Medical Systems (IMSY) German Cancer Research Center (DKFZ) Heidelberg Germany; ^31^ Belgian Royal Academy of Medicine Brussels Belgium; ^32^ Quality and Accreditation Organisation of European Cancer Institutes Brussels Belgium; ^33^ Nova School of Business and Economics Copenhagen Business School & Patient Innovation Frederiksberg Denmark; ^34^ Molecular Oncology FEBS Press Cambridge UK; ^35^ International Agency for Research on Cancer (IARC/WHO) Lyon France; ^36^ INSERM, U1287 and Department of Hematology Gustave Roussy Cancer Center Villejuif France; ^37^ Faculté de Médecine Université Paris‐Saclay Le Kremlin‐Bicêtre France; ^38^ Barcelona Supercomputing Center (BSC) Barcelona Spain; ^39^ ICREA Barcelona Spain; ^40^ Ciencia Viva Lisbon Portugal; ^41^ Karolinska Institutet Stockholm Sweden

**Keywords:** cancer prevention, cancer research, Mission on Cancer, policy

## Abstract

European cancer research stakeholders met in October 2022 in Heidelberg, Germany, at the 5^th^ Gago conference on European Cancer Policy, to discuss the current cancer research and cancer care policy landscape in Europe. Meeting participants highlighted gaps in the existing European programmes focusing on cancer research, including Europe's Beating Cancer Plan (EBCP), the Mission on Cancer (MoC), Understanding Cancer (UNCAN.eu), and the joint action CRANE, and put forward the next priorities, in the form of the Heidelberg Manifesto for cancer research. This meeting report presents all discussions that shed light on how infrastructures can be effectively shaped for translational, prevention, clinical and outcomes cancer research, with a focus on implementation and sustainability and while engaging patients and the public. In addition, we summarize recommendations on how to introduce frameworks for the digitalization of European cancer research. Finally, we discuss what structures, commitment, and resources are needed to establish a collaborative cancer research environment in Europe to achieve the scale required for innovation.

## Graphical abstract

European cancer research stakeholders met in October 2022 at the 5^th^ Gago conference on European Cancer Policy. This meeting report presents all discussions on how infrastructures can be effectively shaped for translational, prevention, clinical and outcomes cancer research, with a focus on implementation and sustainability and while engaging patients and the public.

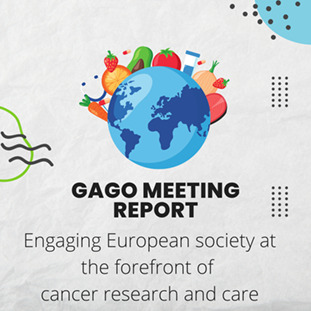

**Abbreviations**
ACC: Alleanza Contro il Cancro
AI: artificial intelligence
CCCs: Comprehensive Cancer Centres
CCE: Cancer Core Europe
CCIs: comprehensive cancer infrastructures
CPE: Cancer Prevention Europe
CSA: coordination and support action
DART: data‐rich clinical trials
EACS: European Academy of Cancer Sciences
EC: European Commission
EBCP: Europe's Beating Cancer Plan
ECPC: European Cancer Patient Coalition
EGA: European Genome‐phenome Archive
EMA: European Medicines Agency
GDI: Genomic Data Infrastructure
JANE: Joint Action on European Networks of Expertise
MoC: Mission on Cancer
OECI: Organisation of European Cancer Institutes
UNCAN.eu: Understanding Cancer
VICC: Variant Interpretation for Cancer Consortium
WPs: work packages

## 1 Introduction

European cancer research stakeholders and policymakers met on 6 October 2022 at the 5^th^ Gago conference on European science policy, which took place at the German Cancer Research Center (DKFZ) in Heidelberg, Germany. The most important action points from this meeting (Table 1) have already been crystallized in the Heidelberg Manifesto on European Cancer Research. This meeting report will be a critical reference point for future discussion about the opportunities and challenges that need to be addressed through new European cancer policy.

**Table 1:** The programme of the 5^th^ Gago Conference on European Science Policy.

Welcome and opening remarks	Michael Baumann, Rosalia Vargas, and Manuel Heitor
Session 1: Emerging developments in European cancer policy Chair: Manuel Heitor (Center for Innovation, Technology and Policy Research, University of Lisbon; and former Minister for Science, Technology and Higher Education, Portugal)	Mario Brandenburg, Parliamentary States Secretary, Ministry of Research and Education, Germany (video message)
	Maria da Graça Carvalho, Member European Parliament (video message)
	Angel Font, “La Caixa” Foundation Barcelona, Spain; President of Cancer and Philanthropy in Europe, Philea
	Francesco De Lorenzo, European Cancer Patient Coalition (ECPC)
	Jeliazko Arabadjiev, Bulgarian Scientific Society of Immuno‐Oncology
Keynote speech Chair: Julio E. Celis, European Academy of Cancer Sciences (EACS) and Danish Cancer Society Research Center (DCRC)	Ulrik Ringborg, European Academy of Cancer Sciences (EACS): Bridging the gaps between research and healthcare delivery
Session 2: Understanding cancer Chair: Eric Solary, Inserm	Eric Solary, Inserm: Introduction to the UNCAN.eu CSA
	Christine Chomienne, Université de Paris: What we do not understand, we cannot address effectively
	Lena Maier‐Hein, DKFZ: Solving the right problem
Roundtable discussion	Elisabete Weiderpass, IARC
	Michael Boutros, DKFZ
	Pedro Oliveira, Patient Innovation, Copenhagen Business School
Session 3: Interconnected high‐quality translational research, clinical and prevention trials, and infrastructures for outcomes research Chair: Anton Berns, EACS	Anton Berns, EACS: Infrastructures for translational research in therapeutics
	Joachim Schüz, IARC: Infrastructures for translational prevention research
	Elena Garralda, VHIO: Clinical trials required for implementation
	Nils Wilking, Karolinska Institutet: Infrastructures for outcomes research
Roundtable discussion	Simon Oberst, OECI
	Stéphane Lejeune, EORTC
	Francoise Meunier, EACS
	Margarida Goulart, Joint Research Centre, European Commission
Session 4: Digital transition in cancer Chair: Marc Lawler, Queen's University Belfast	Alfonso Valencia, Barcelona Super Computing Center: Cancer from a computational perspective
	Fatima Carneiro, University of Porto: Digital Pathology
	Marc Lawler, Queen's University Belfast: Creating a culture of share data
Roundtable discussion	Helena Canhão, Nova Medical School, Lisbon
	Markus Wartenberg, Chair National Patient Council NCT
Session 5: Functional networks of CCCs and other centres: Structuring cancer activities in Member States Chair: Michael Baumann, DKFZ	Michael Baumann, DKFZ (15 min): The German DKTK and NCT model
	Tit Albreht, National Institute of Public Health: Comprehensiveness as the central element in organizing cancer
	Paolo Casali, Istituto Nazionale Tumori (video): Infrastructures in Europe
Roundtable discussion	Rui Henrique, IPO‐Porto
Concluding remarks	Michael Baumann, DKFZ and EACS
	Julio Celis, DCRC and EACS
	Rosalia Vargas, Ciencia Viva

The latest statistics on the impact of cancer on Europe and the world require urgent attention and action by all stakeholders involved in the planning, funding, and management of cancer research. There are almost 4 million new patients and over 1.9 million deaths annually in Europe, with cancer accounting for 25% of deaths in Europe [1]. Population ageing is the main factor contributing to the increase in cancer incidence and mortality, impacting on society and healthcare [2]. Importantly, with new, more effective treatment modalities, cancer survivors have increased to nearly 20 million in Europe [3]. While being an indicator of the advances in cancer research and its translation into the clinic, these numbers sound an alarm bell about the need to adapt European healthcare and regulatory policy for people living beyond cancer as well as cancer patients, as those living beyond cancer have been somewhat neglected in the past from a policy perspective.

The need to transform cancer research strategies was initially highlighted by the EU project EUROCAN+Plus 2005–07, which pointed out the increasing complexity of cancer biology, the necessity to expand translational research by integrating healthcare and research, the establishment of Comprehensive Cancer Centres (CCCs) and the increase of the critical mass regarding patients and infrastructures by institutional collaborations. To analyse and develop cancer research strategies, the European Academy of Cancer Sciences (EACS) was established in 2009. EACS participated in the EurocanPlatform EU‐project 2011–16 with the specific mission of creating sustainability of outcomes, the most important being the Cancer Core Europe (CCE) consortium for therapeutics and Cancer Prevention Europe (see below). EACS actively supported from the start the creation of a Mission on Cancer as an independent organization composed of eminent oncologists and cancer researchers [4, 5, 6, 7, 8].

In this transforming landscape, the European Commission (EC) has recently launched several programmes to tackle some of the major challenges in cancer research and therapeutics/care. These include Europe's Beating Cancer Plan (EBCP), the Mission on Cancer (MoC), Understanding Cancer (UNCAN.eu), which is a MoC implementation programme, the Joint Action on European Networks of Expertise (JANE), and the Joint Action “Network of CCCs: Preparatory activities on creation of National CCCs and EU Networking” (CRANE) (Table 2). In addition, the Lancet Oncology European Groundshot Commission recently published 12 recommendations, which, if acted upon, would reimagine a cancer research agenda for Europe [9]. The participants of the 5^th^ Gago conference – leaders in basic, translational, and clinical cancer research and representatives of cancer research institutes, EU programme boards, European cancer patients, and the European Academy of Cancer Sciences (EACS) – discussed existing European cancer research frameworks, examined the gaps that need to be bridged, and placed considerable emphasis on strategies that place patients at the forefront of European cancer research and care. Moreover, they discussed how to prioritize innovation, identified lingering challenges, for example, in the context of governance and resources for European cancer research structures, and discussed how pan‐European collaboration and public engagement could be achieved.

**Table 2:** A list of EU actions aimed at supporting cancer research and care.

Europe's Beating Cancer Plan (EBCP)	An EU programme launched in 2021 that aims to address key open issues related to cancer prevention, early cancer detection, cancer diagnosis and treatment, and quality of life for cancer patients and cancer survivors [43]
The Mission on Cancer (MoC)	An ambitious portfolio of EU actions (including policy, funding, and, even, legislative initiatives) aiming at improving cancer prevention, diagnosis and care by 2030, with the active involvement of multidisciplinary cancer researchers and citizens in newly defined infrastructures [44]
Understanding Cancer (UNCAN.eu)	A European initiative aimed at building a federated cancer research data hub that is connected to a research agenda
4.UNCAN‐eu	A 15‐month action aimed at coordinating and supporting the preparation of the UNCAN.eu platform
Joint Action on European Networks of Expertise (JANE)	A Joint Action with the aim to create or upgrade CCCs in EU Member States, and build a network among existing and newly established CCCs
Joint Action “Network of CCCs: Preparatory activities on creation of National CCCs and EU Networking (CRANE)	A Joint Action with the aim to establish by 2025 an EU Network linking national CCCs
The Lancet Oncology European Cancer Groundshot Commission	The most comprehensive analysis to date of cancer research activity in Europe, underpinning a series of 12 Recommendations to reimagine cancer research and its implementation across Europe
Cancer Prevention Europe (CPE)	A consortium of European organizations that aims to reduce morbidity and mortality from cancer in European populations through prevention and earlier diagnosis
EU for Health (EU4H)	An EU programme that was initiated in response to the COVID‐19 pandemic to reinforce healthcare systems in the EU
Personalized Cancer Medicine for all EU citizens (PCM4EU)	A project that started in early 2023 under the umbrella of EBCP to facilitate the implementation of molecular cancer diagnostics for precision oncology
Cancer Core Europe (CCE)	A consortium of European cancer research centres that aims towards the rapid translation of early discoveries to the clinic
Data Analysis and Real World Interrogation Network (Darwin EU)	A coordination centre established by European Medicines Agency (EMA) and European medicines regulatory network to provide timely and reliable evidence on the use, safety and effectiveness of medicines for human use, including vaccines, from real‐world healthcare databases across the European Union (EU). DARWIN EU will connect the European medicines regulatory network to the European Commission's European Health Data Space (EHDS) [28]
European Health Data Space (EHDS)	An EU framework that aims at providing increased digital access to and control of electronic personal health data of EU citizens, at national level and EU‐wide. EHDS supports a genuine single market for electronic health record systems, relevant medical devices and high‐risk AI systems and provides the framework for consistent, trustworthy and efficient use of health data for research, innovation, policy‐making and regulatory activities [45]. The EHDS pilots for secondary use include a cancer genomic use case
Genomic Data Infrastructure (GDI)	A Europe‐wide project enabling access to genomic and related phenotypic and clinical data [46]
ELIXIR	The European bioinformatics infrastructure for biological data access
Genome Data Alliance	European Project for the implementation of the B1MG developments and recommendations in the signatory countries
EOSC4Cancer	EU project aiming to build the federated infrastructure for secondary use of cancer data
European Genome‐phenome Archive (EGA)	The main repository of genomic data (omic data, in general), including the version for data federation “Federated EGA”
Global Alliance for Genomics and Health (GA4GH)	The main global organization for discussion and provision of standards for genome analysis in medical context
DATA‐CAN	The UK's Health Data Research Hub that was launched in the UK in 2019
German Cancer Consortium (DKTK)	A National Consortium bringing together over 20 institutes and hospitals to support translational cancer research
Joint Action CanCon	An EU Joint Action that resulted in the publication in 2017 of a Guide on how to control the cancer burden in Europe
CCI4EU	An EU Coordination and Support Action to build capacity in Comprehensive Cancer Infrastructures (CCIs), with a special focus on CCIs with less maturity
ECHoS	An EU Action for the creation and development of National Cancer Mission Hubs capable of implementing the EU Cancer Mission at national, regional and local levels

Discussions at the 5^th^ Gago meeting echoed the words of Prof. José Mariano Gago:

Considerable time should be devoted to setting up a dialogue in each Member State on the issue of human resources for science, engineering, and technology, helping policymakers understand what is required, and building bridges between national and European actors. Dialogue between industrial and academic organizations in Europe should also be pursued. (European Commission “Europe needs more scientists” meeting, Brussels, 2004)

In this Meeting Report, we aim to highlight these discussions and how they resulted in the Heidelberg Manifesto.

## 2 Emerging developments in European cancer policy

Michael Baumann, Director of the German Cancer Research Center (DKFZ), Rosalia Vargas, President of the Portuguese National Agency for Scientific and Technological Culture Ciência Viva, and Manuel Heitor, former Portuguese Minister of Science, Technology and Higher Education, opened the Conference. Their brief welcome note was followed by video messages of support by the Parliamentary State Secretary to the Federal Minister of Research and Education in Germany, Mario Brandenburg, who presented cancer research infrastructures as a key priority in Germany and Europe, and by the European Parliament member, Maria de Graça Carvalho, who committed to pursuing innovation and breakthrough for cancer science policy, on the steps of her predecessor, Prof. José Mariano Gago. The keynote speech by Ulrik Ringborg, Director of Cancer Center Karolinska, highlighted the various gaps in the cancer research continuum and their impact on translational research [8].

Following talks by Eric Solary (INSERM, France) and Christine Chomienne (Université de Paris) provided an update on the progress of the European programmes on cancer research, UNCAN, and MoC. Some discussions during this first part of the meeting and the roundtable contributions of Anton Berns (NKI), Elisabete Weiderpass (IARC), and Michael Boutros (DKFZ) helped to highlight convergence and divergence points between current European programmes supporting cancer research and the EACS targets for cancer research (Box 1).

Box 1 **EACS targets for cancer research**

The EACS targets for cancer research include:

- Decrease incidence by primary prevention and screening strategies for detection and removal of relevant precursor lesions
- Early detection of invasive cancer with and without metastatic properties
- Increase cure – precision local and regional therapy (surgery and radiation therapy), precision systemic treatment (anticancer agents, immune‐oncology)
- Increase survival when cure is not possible
- Improve health‐related quality of life
- Cost‐effective cancer therapeutics, cancer care and prevention

### 2.1 Setting the frame for innovation in European cancer policy

According to Elisabete Weiderpass, the costs of cancer care will soon become unbearable. Cancer premature mortality translates into a substantial economic and societal burden, which amounted to almost €200 billion in Europe in 2018, consisting of both direct costs, that is, cancer‐specific health expenditures including expenditure on drugs, and indirect costs, as, for example, costs resulting from premature mortality, morbidity, and informal care [10]. The total value of productivity lost due to early cancer mortality in 2018 was estimated at €104.6 billion in Europe [11]. Largely related to population ageing, the number of new cancer cases among older adults (aged 65+) in Europe is expected to double by 2035. These trends will place increasing pressure on government fiscal finances and strain healthcare budgets [11]. In this regard, the European cancer policy scene already features committed politicians pushing the cancer agenda. In such a mature political setting, cancer researchers and care specialists are now called to develop a plan that exemplifies prioritizing and addressing the most urgent cancer research and care needs in Europe.

In his keynote speech, Ulrik Ringborg delivered the message that integration between research and health care is under development in Europe, with increasing numbers of CCCs being launched. The healthcare sector is preserved as key infrastructure and becomes gradually connected to translational cancer research.

In an effort initiated through the EUROCAN Plus EU project 2005–07, key stakeholders of European cancer research have defined the European cancer research continuum [12]. As noted by Ulrik Ringborg, we are approaching a European cancer research ecosystem that encompasses all key elements of the cancer research continuum. The research continuum for cancer therapeutics involves the following components: basic and preclinical research with development of proofs of concept for clinical trials; early clinical trials, next‐generation clinical trials, practice‐changing clinical trials/treatment optimization studies followed by implementation research; outcomes research for the assessment of clinical effectiveness and health economics research. Health‐related quality of life and survivorship research are additional important research areas integrated with therapeutic research. A similar research continuum represents cancer prevention research, starting with identification of a prevention context as an outcome of basic and preclinical research for further intervention research before implementation in a prevention programme. For prevention as well as for therapeutics, relevant outcomes and health economics research are missing elements. Effective translational research is achieved when gaps between the different components of the research continuum are bridged. Reverse translational research brings clinical and prevention problems back to basic and preclinical research for biological explanations. (Table 3). Essential to bringing further progress to all these aspects of research aiming at personalized/precision cancer medicine is to address the critical mass problem, namely ensuring a critical mass of infrastructures, resources, knowledge, specialists, patients, and related biological and clinical data, to achieve new breakthroughs. This requires international collaborations.

**Table 3:** The evolving cancer research ecosystem.

Early translational research sets the agenda for the clinical and prevention research. Basic biological and technological research is highly innovative, resulting in a larger number of new innovations than what can be implemented in the healthcare and prevention. Prioritize basic research innovations of potential interest for therapeutics care and prevention. Increased focus on preclinical research is a need to prime the early clinical and prevention research for proof‐of‐concept clinical trials or prevention contexts
Clinical trials also of increasing complexity Early clinical trials – proof‐of‐concept trials. Next‐generation clinical trials. Technologies for stratification of patients for treatment. Combinations of anticancer agents and treatment modalities are unmet needs. How to define criteria of clinical efficacy, clinical effectiveness, and cost‐effectiveness for recommendations for the healthcare and organizations responsible for prevention. Long‐term follow‐up of clinical innovations for assessment of health‐related quality of life (survivorship research)
Prevention Primary prevention Secondary prevention: early detection of precursor lesions through prevention screening Tertiary prevention: decrease of morbidity and disability after therapy
Health‐related quality of life Supportive care Psycho‐social oncology Tertiary prevention Rehabilitation Palliative oncology and end‐of‐life care Survivorship Control of acute and late side effects Technologies to predict side effects as a component of precision cancer medicine
Outcomes research Outcomes of specific innovations Outcomes of cancer treatment/care and prevention Outcomes research for health economics research Goals of the mission on cancer: incidence and cancer mortality standardized to the European population; relative survival and health‐related quality of life [42]
Reverse translational research Identification of needs observed in clinical trials and cancer therapeutics/care for further biological research Biobanks of repeated biopsies (fine needle aspiration and liquid biopsies) Improvement of adaptive medical treatment
The critical mass problem: CCCs and consortia of CCCs Outreach responsibility of CCCs Paediatric oncology, geriatric oncology Specific diagnostic and treatment infrastructures International collaborations to mitigate inequalities
The stakeholder problem Cancer researchers Healthcare and prevention professionals Patients as research partners Decision makers on research budgets, grant agencies Industry Political level Media, general public

It is imperative that the critical mass in terms of research and care specialists and infrastructures is ensured in every European country, as highlighted by Jelazko Arabadjiev, President of the Bulgarian Scientific Society of Immuno‐oncology and member of the EU MoC board. To achieve this, we must address inequalities and stimulate concerted efforts to avoid other obstacles, including the brake exerted on progress by the COVID‐19 pandemic. Jelazko Arabadjiev also emphasized issues such as making the field attractive to young researchers and cancer treatment and care specialists, covering the cost of open‐access publications in lower income countries, and ensuring the latest therapies, including immunotherapies, are kept in pace with research in lower income countries.

### 2.2 An update on EU Mission on Cancer

It is within the above cancer research and policy landscape that the EU launched the MoC implementation plan on 29 September 2021. The MoC sets the ambitious goal of saving more than 3 million lives by 2030, with cancer survivors living longer and with a better quality of life. MoC defines four areas of action: **prevention**; **diagnostics** and **treatment** of cancer; **quality of life** of cancer patients, cancer survivors and their families and carers; and **equitable access** to all the above across Europe. Importantly, within the context of the MoC implementation plan, up to €2 billion will be invested to gain a better understanding of cancer. In detail, it is sought to understand: why certain gender‐, age‐ or other population groups are at higher risk for cancer; what underlies resistance to cancer therapies; and what are the bottlenecks during the design of impactful and cost‐effective cancer prevention and screening programmes, devices, diagnostic tests, treatment and care solutions adapted to patients. Importantly, the MoC implementation plan sets the objective of launching the UNCAN.eu platform by 2023 (see also Section 2.3).

Christine Chomienne, Vice‐Chair of the MoC Board, gave a comprehensive overview of the Mission, its objectives, and the next steps for implementation. The definition of missions in the context of Horizon Europe was shaped by the 2018 and 2019 reports of Mariana Mazzucato [13, 14]. For their implementation to be successful, Horizon missions should, according to Mazzucato's recommendations, engage citizens in co‐designing, co‐creating, co‐implementing, and co‐assessing the outcomes, introduce innovation in public sector capabilities and rely on mission‐oriented financial leverage and crowding‐in forms of funding.

The MoC Board, formed between September 2019 and September 2020, includes experts from cancer research, policy‐, care‐, other specialists, and patients. The MoC Board has since, with the additional support of assembly members, including a wider network of experts, been involved in citizen engagement meetings including stakeholder events and meetings with two EU‐wide focus groups. Board members act as ambassadors, meeting with all European Ministers of research, members of the European Parliament, the BECA committee, citizen engagement groups, and other national and European stakeholders, often consulting published input by these stakeholders.

### 2.3 Implementation of Understanding Cancer

Following the recommendations of MoC and EBCP, the UNCAN.eu programme was designed to introduce a new level of investment in innovative research, including high‐potential and high‐risk projects. The UNCAN.eu implementation plan, which combines ambitious objectives with specifically outlined interventions, is expected to deliver a cancer research platform with European‐wide value. A 15‐month coordination and support action (CSA, collectively referred to as 4.UNCAN.eu) was launched in September 2022 to design, through six work packages (WPs) and four workshops, the blueprint for the establishment of the UNCAN.eu platform.

Eric Solary introduced the structure of the 4.UNCAN.eu initiative, led by INSERM and involving 29 core, advisory, and consultation partners, all working to define specific interventions, a concrete investment strategy, and selective progress indicators. Partners are engaged in six WPs to deliver suggestions on two key objectives within 15 months. These WPs will work to design a federated cancer research hub and to generate a series of “research use cases”, that is, cross‐border and interdisciplinary research programmes that will feed research data into UNCAN.eu. The two key objectives will be addressed while ensuring organizational, logistical, financial, and structural feasibility for UNCAN.eu, emphasizing sustainability. WP1 (coordinated by INSERM) is focused on the coordination of the above efforts, WP2 (coordinated by Ciberonc and Alleanza Contro il Cancro (ACC)) will define research use cases, WP3 (coordinated by the DKFZ and INSERM) will develop a framework for the cancer research data hub, WP4 (coordinated by ECPC and Childhood Cancer International – Europe (CCI‐Europe)) will work to involve patients and citizens into planning and implementation, WP5 (coordinated by ACC and DKFZ) will take over the organization of the programme, and WP6 (coordinated by Hungarian National Institute of Oncology (NIO) and NKI ONCODE) will aim at tackling inequities among EU member states.

As highlighted by Solary, as soon as UNCAN.eu becomes a federated cancer research hub, it will host large‐scale data collected from patients and generated by experimental models. Data will span over six areas of cancer research: (a) cancer prevention; (b) early cancer diagnosis; (c) tackling cancer therapy resistance; (d) cancer survivorship; (e) paediatric cancer; and (f) cancer and ageing. This hub will not require the *de novo* formation of research structures. It will rather involve existing research infrastructures (the so‐called ERICs), including basic and translational research entities, comprehensive cancer centres, and cancer registries, all providing a multitude of data, including patient biological and clinical data, patient images, patient lifestyle data from patient‐derived models, etc. As such, and given the tight timeframe of the 4.UNCAN.eu CSA, it will be imperative to work towards increasing the visibility of the general scope among European cancer researchers and providing concrete solutions on organizational aspects that will help align all existing infrastructures with the federated scheme.

Michael Boutros, who leads the WP3 on developing a federated cancer research hub blueprint, discussed how UNcan.eu may be viewed as a European infrastructure or a virtual institute. The key challenge in next months' efforts to define the blueprint is to identify an appropriate governance model to ensure sustainability and a long‐term impact. The 4.UNCAN‐eu project will discuss with stakeholders good examples of governance and organization of infrastructures under the umbrella of a hub that aims at accelerating research and training a new generation of cancer scientists while remaining in close contact with societal needs for citizen involvement and research equality.

Importantly, for UNCAN.eu to fulfil the long‐term goals of improving cancer care, cancer survivorship and quality of life, and to tackle inequalities in cancer research, while also involving patients and citizens in the planning of such a future European cancer research platform, an additional challenge will be to define the “research use cases”. Specifically, the research programmes initiated through UNCAN.eu must be defined in such a way so that they efficiently and quickly fill the platform with new and diverse data, making it indispensable to cancer researchers. It is expected that if “research use cases” are designed so that they can attract the researchers' interest, researchers will become involved in UNCAN.eu early on, ensuring a bottom‐up approach. In addition, it is hoped that with the support of data science and efficient data app will be built to bridge the gap between basic and clinical research data and infrastructures. Ultimately, success will depend on extended communication efforts to engage the next generation of researchers and clinicians in UNCAN.eu.

### 2.4 Priorities for European cancer policy: Divergence between EC calls and policy goals

In the above landscape that combines the pressing societal need for battling cancer at multiple levels and the responding EC calls, all aimed at addressing key cancer research and care gaps under a tight timeframe, it is imperative to ensure effective prioritization, careful design and, ultimately, engagement of all parties involved. As such, discussions at the 5^th^ Gago Conference have helped to highlight running EC calls of considerable mass, but also bridge policy goals and political actions, while flagging action points and next steps. Before discussing specific requirements and priorities for supporting cancer research, cancer prevention, cancer care, and cancer survivorship through specific initiatives, meeting participants summarized **four main action points** emerging through discussions at the meeting.

**Action point 1: Tackle inequalities across Europe**, as brought forward by Jeliazko Arabadjiev (who discussed cancer research and care in Eastern Europe) and Françoise Meunier (who discussed the legal framework for cancer survivorship; see also Section 3).

**Action point 2: Bridge the gaps between research and healthcare delivery,** as highlighted in the keynote speech by Ulrik Ringborg. The clinical efficacy of practice‐changing clinical trials and treatment optimization studies is required for implementation research assessing clinical effectiveness and cost‐effectiveness. With the increasing costs for cancer therapeutics and care, it is surprising that outcomes and health economics research still are missing elements. This is also the case for cancer prevention programmes. An important emerging bottleneck towards these aims is the shortage in personnel (doctors, nurses and researchers) that has fast become apparent during the recent COVID‐19 pandemic and is expected to deteriorate further in the following decades, as highlighted by Anton Berns.

**Action point 3: Improve health economics** by complementing the existing MoC plan. Further emphasis should be placed on health economics and implementation science [9]. Moreover, efficient patient‐handling structures that support the management of side effects or non‐responders in the context of clinical trials are needed. Further research also needs to be carried out in cancer survivorship. Finally, a clearer focus on health economics for prevention is required.

**Action point 4: Promote cancer policy scope and organization** by better integrating the cancer research community and increasing their contact with policymakers and politicians. A bottom‐up process will be important in this respect, and EACS can contribute towards this aim; as highlighted by Michael Baumann, involving all stakeholders will facilitate cancer policy and cancer research design for the EU bodies.

Complementary to the above four action points, the urgent need to involve patients and cancer survivors, their families, and the general public in the above‐outlined complex cancer policy landscape of Europe has already been highlighted through current EC programmes, publications, policy initiatives, and political movements. A patient‐centred scope and the importance of public engagement are viewed as key components in all four action points outlined, and ways to sustain and further develop them are, thus, examined separately in the following section.

## 3 Patient‐centred scope and public engagement with a focus on scientific culture

### 3.1 Educational platforms for advocacy patients and the potential role of science centres

As noted by the President of the European Cancer Patient Coalition (ECPC), Francesco de Lorenzo, patient organizations gradually become increasingly involved in research through advocacy. In the context of their recent trio EU council presidency, Germany, Portugal, and Slovenia adopted principles of successful patient involvement. ECPC is a link between stakeholders, always focusing on patient needs, ethics, and sustainability. It strongly advocates for partnership between researchers and patients, allowing patients to contribute their experience towards effectively translating research into clinical practice.

While advocacy patients are systematically involved as active partners or co‐researchers, holding a fair share of decision‐making power, ECPC works to develop educational platforms to guide their participation in cancer research. ESO learning and IARC repository platforms will be used to develop educational materials to inform patients nationally. ECPC has also been involved in driving personalized/precision medicine and has produced a booklet and a leaflet that explain these concepts and their practical applications in lay language and indicate potential questions that a patient may choose to ask their doctor.

Rosalia Vargas and Pedro Russo from Ciência Viva emphasized that cancer awareness programmes, ranging from science education to public campaigns, must be strongly supported to foster a general understanding of cancer research, prevention and outcomes. Science centres should play a relevant role by involving cancer researchers, physicians, patient representatives, and science communicators. Increased cancer awareness throughout the European population is key to early detection. Better health‐seeking behaviours, particularly among Europe's most vulnerable countries, should be recommended.

In the context of MoC and in collaboration with the Organisation of European Cancer Institutes (OECI), ECPC will develop an educational module to explain to patients how they can contribute and their roles within CCC infrastructures. Presently, ECPC is involved in 26 research programmes (big data and personalized medicine, knowledge sharing networks, patients and caregivers, palliative care, treatment, and European policy (UNCAN)). In the future, patient advocates and citizens will be involved in the governance of new organizations such as UNCAN.eu.

### 3.2 Public engagement through philanthropy and user‐driven innovation

Angel Font, Corporate Director of Scientific Research at La Caixa Foundation and Chair of the Philanthropy Europe Association (Philea), indicated that philanthropic organizations may act as catalysts in efforts to engage the public in all aspects of cancer research and bring together the public and private sector to ensure a fast pace of progress. While public institutions offer suitable infrastructures to foster innovation throughout the cancer research continuum and the private sector remains financially sustainable, philanthropic organizations may be considered as a safe common space to boost high‐risk projects and facilitate collaboration. To achieve this aim, Angel Font stressed the need for cooperation between charities and the implementation of higher level structures that will facilitate the exchange and coordination of actions.

Pedro Oliveira, research leader on “user innovation” and professor at the Copenhagen Business School, presented the patient‐innovation.com platform as a good example of how patients can be integrated through specific structures and funds in cancer research healthcare innovation. The introduced not‐for‐profit, open platform supports patients while they propose and develop solutions to disease‐related challenges throughout all stages: from an initial research hypothesis to commercialization.

### 3.3 Research on survivorship care and cancer cure

In addition to shaping research directions based on their needs and experiences, with the ultimate aim to improve cancer care and quality of life, cancer patients and survivors must determine priorities towards shaping healthcare, as well as the social and legal frames that can support current and future cancer survivorship and cure figures. Francesco De Lorenzo, noted that about 5% of the individuals in several European countries live after a cancer diagnosis (for example, 3.6 million in Italy). This percentage is estimated to grow by approximately 3% per year, and a large proportion of diagnosed individuals (indicatively, 24% of the cancer patients in Italy and 29% in the USA) are alive 15 or more years after diagnosis. Patients living after a cancer diagnosis include those under treatment, those who are disease‐free but remain at excess risk of recurrence, those who have treatment‐related chronic conditions, and those who have the same life expectancy as the general population (that is, those that are cured) [3]. While progress in cancer research, early cancer detection and clinical care are expected to further increase the numbers of cancer survivors across Europe, it is imperative to work on how cancer survivors' needs can be best accommodated through European cancer policy initiatives.

Cancer survivorship requires long‐term follow‐up examinations, with considerable consequences for the individual, the healthcare system, and society. Robust healthcare structures will be necessary to ensure that related patient needs are effectively accommodated. As a result, interdisciplinary research on cancer survivorship, which is presently quite limited, will be crucial to help build structures that serve the needs of cancer survivors. An important component of the above is specific research programmes for children, adolescents, and young adult survivors. Equally, Francesco De Lorenzo stressed the lack of detailed research on cancer survivorship support for patients and patient empowerment.

A cancer survivorship research field requiring particular attention is that of legal research. We presently lack sufficient knowledge of the general stigma associated with cancer and the financial consequences that cancer survivors may often have to face. As highlighted by Francesco De Lorenzo, this research gap also be translated into a lack of concerted political and legal actions. As further elaborated by Francoise Meunier, this research gap may also be linked with a lack of concerted political and legal actions towards Europe‐wide implementation of the “right to be forgotten”, that would shield cancer survivors against any financial discriminations stemming from their patient record history [15].

Although implemented through legal frames in six European countries, namely in France, Belgium, Luxembourg, The Netherlands, Portugal, and Romania, the “right to be forgotten” must be introduced more widely in the context of MoC. Long‐term data collection is needed to support the adoption of a solid legislation frame across the EU. The code of conduct to be delivered by the EU in 2024 is an encouraging first step, but a solid legal framework will be essential in the future, particularly as it has shown feasibility and effectiveness in five EU Member States. This will also ensure that inequities across EU member states are addressed, with cancer survivors having equal financial and legal rights throughout Europe. As Françoise Meunier concluded, Europe needs to deliver a strong positive message about cancer survivors having equal rights as any other European citizen [15].

## 4 Scale and innovation through interconnected high‐quality infrastructures for translational research, clinical and prevention trials, and outcomes research

After having highlighted priorities in the current cancer policy landscape in Europe, the second part of the conference focused on the specifics of policy plan implementation. In a session joined by Anton Berns, Joachim Schüz (IARC), Elena Garralda (VHIO), and Nils Wilking (Karolinska Institutet), the specific requirement for infrastructures to promote translational research for cancer therapy and cancer prevention, clinical trials, and outcomes research were discussed in detail. A concluding roundtable discussion with Simon Oberst (OECI), Stephane Lejeune (EORTC), Françoise Meunier (EACS), and Margarida Goulart (Joint Research Centre of the EC) helped to highlight common themes in efforts to further develop and connect such infrastructures, as reported below.

### 4.1 Cancer prevention research infrastructures

Joachim Schüz, head of the Environment and Lifestyle Epidemiology Branch of IARC and chair of Cancer Prevention Europe (CPE) – a consortium of key European institutions focusing on cancer prevention research – discussed the requirements for infrastructures for translational research focusing on cancer prevention in Europe [16]. Over 40% of new cancer cases are preventable through primary prevention interventions. Such interventions could target smoking (responsible for almost half of all preventable cancers), obesity and physical inactivity, unhealthy diet, alcohol consumption, infections, ionizing and ultraviolet radiation, and occupational or environmental factors. For a smaller fraction of cancer diagnoses, causes have been identified but appear not to be preventable, such as genetic factors or exposure to naturally occurring agents that cannot be eliminated, while for the other half of new cancer diagnoses potential prevention approaches and their impact remain unknown [17]. This highlights the great knowledge gap that must be addressed by the three main components of prevention research: continued aetiological research, seeking to identify causes of cancer; intervention research, investigating the most effective interventions to prevent cancers with known and modifiable risk factors; and implementation research, focusing on how effective interventions can be tailored to achieve cost‐effectiveness and fit the cultural, behavioural, and social circumstances of the target populations.

Infrastructures for prevention research, such as CPE, will need to equally address the above three research directions, as well as incorporate outcomes research for all three components to monitor progress. Importantly, while investing on these infrastructures, policymakers and European government bodies must consider that this is essentially a long‐term investment, as even rigorous implementation of cancer prevention interventions will show significant benefits only after a considerable period of time. This is due to the slow development of cancer diseases, meaning that most of the cancer cases occurring in the upcoming three decades are due to exposures that occurred in the past: an example is asbestos‐related mesothelioma in Germany, where the peak of asbestos‐related mesothelioma deaths was reached only between 2020 and 2022 after banning asbestos three decades earlier [18]. Involvement of or cooperation with the existing networks of CPE, as well as IARC/WHO, the WHO EURO Regional Office, the Joint Research Center (JRC) of the EC and the Association of European Cancer Leagues (ECL), will help ensure both effectiveness and timeliness of any future European initiatives focusing on cancer prevention. This point was reinforced by Margarida Goulart from the JRC, who highlighted the role of JRC in supporting integration of translational research data to policy and knowledge dissemination that can help tackle information overload and accelerate implementation.

### 4.2 Clinical trial infrastructures

Current and future attempts to build or reinforce cross‐border infrastructures for cancer therapy‐oriented clinical trials of smaller or larger scale may also learn from the experience of existing European consortia – and this was a key message delivered by Elena Garralda, Director of the Early Drug Development Unit of VHIO, Barcelona, Spain. Elena Garralda emphasized the exponential increase of cancer patients receiving and responding to targeted therapies following personalized cancer genomics analyses in the US [19]. These increased numbers of patients under targeted treatments reflect the improvement of tools for studying specific genomic alterations that have shifted tumour classification away from being based solely on tumour type and histology.

Clinical trials aiming to achieve broader implementation of genomic analyses and personalized targeted therapies still need to overcome a series of bottlenecks (Table 4). Importantly, the wide availability of cancer genomic data is useless without a framework allowing for interpretation and effective translation into clinical practice. Ongoing efforts already aim at overcoming the above‐listed limitations. For example, ESCAT (ESMO Scale for Clinical Actionability of molecular Targets) is a framework aiming to rank genomic alterations as targets for cancer precision medicine [20]. Moreover, a network of hospitals and cancer centres, initially involving institutions in the Netherlands, Sweden, Norway, Denmark, and Finland, and later including cancer centres in France, Poland, Germany, Italy, Portugal, Spain, Lithuania, Croatia, and Estonia, implemented the Drug Rediscovery Protocol (DRUP) trial design to define early signs of responsiveness to approved drugs beyond their labels since 2010 [21]. In addition, the EU for Health (EU4H) programme initiated in 2021 as a response to challenges posed by the COVID‐19 pandemic and investing in reinforcing national health systems until 2027 includes the Personalized Cancer Medicine for all EU citizens (PCM4EU) component. PCM4EU, led by Hans Gelderbloom, aspires to facilitate the implementation of molecular diagnostics and molecular tumour boards and to implement DRUP‐like trials in the EU. This can be achieved while pooling result data from such trials to facilitate the implementation and dissemination of personalized cancer treatments. PCM4EU also seeks to ensure equitable access to such clinical trials and address gaps in patient education.

**Table 4:** Bottlenecks for the implementation of NGS‐based personalized targeted therapies.

Stages	Challenges
Patient education	Inform patients about available options, their promises, and limitations
Sample acquisition	Improve sample yield and quality
	Ensure representative sampling
	Account for tumour heterogeneity and clonal evolution
NGS assays	Enable analytical validation
	Improve cost effectiveness
	Ensure broad availability and scalability
Bioinformatic analyses	Ensure standardization
	Address requirements for manual curation
Reporting	Standardize target prioritization
	Consider clinical relevance
Tumour boards	Aim at scalability
	Detach from the academic research setting
Personalized targeted therapy	Ensure availability of the suitable drug

Finally, Dr Garralda highlighted progress made by Cancer Core Europe (CCE), an independent consortium of major cancer centres in Europe that have worked together to create a multi‐site European Cancer Institute, where joint translation and clinical research is performed, with a special focus on next‐generation clinical trials and precision medicine. The creation of large, shared databases through CCE is expected to accelerate cancer diagnosis, clinical decisions, and outcomes research throughout Europe, even when small subsets of cancer patients are targeted. The Basket of Baskets (BoB) multimodal clinical trial design and the CCE Molecular Tumour Board exemplify how the above can be achieved [22, 23] and have further developed with the recent funding from the EU's Horizon 2020 research and innovation programme to build data‐rich clinical trials (DART). The DART project was launched in February 2021, with the aim to develop digital tools for trial management and clinical decision‐making. DART is expected to improve clinical trial methodology through the introduction of new statistical designs. Moreover, it aims at integrating more accurate imaging and molecular biomarkers of treatment responses into a new generation of clinical trials, while also ensuring patient empowerment and involvement or engagement (Table 2).

Valuable lessons can be learned from the structure and governance of a sustainable cross‐border cancer research infrastructure, such as CCE. CCE already delivers high volume of clinical activity, with 60 000 new patients available for the CCE trials annually, and with data from 250 000 to 300 000 treated patients being registered and analysed per year. The commitment and coordination of cancer centres and universities across Europe to deliver basic, translational, and clinical research programmes, while maintaining an investigator‐initiated trial culture could be informative and supportive of any future attempts to further expand cancer research infrastructures. Importantly, the development of CCE to this current structure took a considerable amount of time (7 years) and lacked initial EU funding. This indicates that future concerted efforts involving existing structures and know‐how, as well as financial support from the EU have the potential to further accelerate the formation of similar networks across Europe.

### 4.3 Outcomes research infrastructures

Nils Wilking, Karolinska Institutet, Stockholm, Sweden, stressed the necessity for connecting outcomes research infrastructures to a European cancer research network. When most new therapies get into the clinics, they are backed with no or limited outcomes research relying on real‐world data. However, while clinical trials are designed to check for efficacy, outcomes research is needed to assess benefits for larger patient cohorts and cost‐effectiveness. Whereas clinical trials test new interventions only under specific controlled conditions, outcomes research can provide information about how patients respond to new interventions depending on clinical practice and comorbidities. Importantly, clinical trials usually only provide insights into relative prognostic risk and progression‐free survival, whereas outcomes research may shed light onto overall survival and quality of life. Data stemming from clinical trials are more homogeneous as compared to the real‐life clinical setting, and it is imperative that real‐world data on outcomes associated with new cancer medicines are analysed to inform future clinical decisions [24]. Priorities in outcomes research are, thus, to ensure the quality of real‐world data and create structures for systematic collection of data on age, gender, diagnosis (with index dates), stage of disease, comorbidities, outpatient and inpatient care, treatment, treatment outcomes (including times to treatment failure), and date of death. In addition, patient‐reported experience measures must be introduced, and direct costs of treatment must be registered. Importantly, real‐world data are needed equally from cured patients and patients with advanced disease.

Like other infrastructures, outcomes research infrastructures do not need to be invented from scratch. Existing real‐world data archives, such as the National Cancer Diagnosis Audits in the UK, AIFA registries in Italy [25], Open Comparisons in Sweden [26], CancerLinQ, a subsidiary of ASCO [27], and Flathealth (flatiron.com/real‐world‐evidence), can be integrated into a pan‐European outcomes research network.

Importantly, the European Medicines Agency (EMA) and European Medicines Regulatory Network have recently established the coordination centre Darwin EU that will work to build a data network for high‐quality real‐world data on the safety and effectiveness of medicines. Darwin EU is expected to enter full operation by 2024 [28]. In parallel, the OECI has recently installed the Cancer Outcomes Research working group. The main aim of OECI is to establish and coordinate a network of clinical cancer centres adopting common procedures for the systematic collection and sharing of data pertinent to patient‐reported outcome measures and patient‐reported experience measures (https://www.oeci.eu/WG.aspx?id=13&group=1).

While EMA and FDA regulators call for collaboration to integrate real‐world evidence into regulatory decision‐making, the EC launched the European Health Data Space in May 2022. This will allow for the deposition of patient data in a common European format and create a legal framework to regulate data usage for research, regulatory or policy‐making purposes, subject to data control by individual citizens. While a European outcomes research infrastructure is in the works, attention will be required from the MoC to support its development further.

### 4.4 Next steps towards high‐quality infrastructures

The current understanding of the EU cancer research programmes is, according to Anton Berns, that EBCP will focus on implementing existing and new knowledge in clinical practice, whereas MoC, including the UNCAN.eu platform, will be oriented towards all forms of cancer research: basic, translational, clinical, prevention and outcomes research.

While shaping the critical infrastructures for the above, two pressing priorities emerge. First, a quality control mechanism must be established to ensure the quality of emerging new data and suitability for clinical implementation. Second, sustainability must be considered when developing cross‐border infrastructures to support the cancer research continuum. These infrastructures will be combined into a sustainable entity with well‐defined scientific goals and robust governance. As this need cannot fit within or be accommodated by the current funding schemes of the EU, there is a need for organizational structures that differ from those of the regular EU funding routes.

In detail, such organizational structures may involve platform support to foster close collaboration between centres. Contrary to how some current EU funding schemes work, more is needed to support high‐risk projects, and concerted efforts are required to prove that such projects are intelligent and target oriented. Importantly, Dr Berns stressed the need to install programmes that directly support innovative PI‐led projects, and not necessarily aggregates, while also emphasizing basic research. Equally, Dr Berns stressed the need to fund PI‐initiated innovative clinical trials at phases I and II.

Specific areas requiring attention are the areas of health‐related quality of life, psycho‐social and socio‐economic research, as well as implementation research and outcomes research integrated with health economics. While working to connect and develop research infrastructures, it will also be important to recruit a board of experts to advise on legal measures, for example measures pertaining to cancer prevention. In addition, overarching potential issues across the cancer research continuum must be addressed while articulating the next MoC calls. To ensure increased engagement of researchers from across all respective fields, MoC calls need to be made less diffuse. Moreover, initiatives to re‐invent existing infrastructures, such as CCCs and other European networks, should be avoided. Importantly, MoC calls may need to be tailored to become more appealing and relevant to institutes.

As a result, EACS emphasized the need to directly involve representatives of the respective fields in efforts to articulate future MoC calls so that they can effectively target the appropriate audience. In addition, it is crucial to involve all stakeholders in a sound and transparent governance scheme spanning the various infrastructures. In such attempts, it will be key to maintain networks simple and sustainable, as in the examples of CCCs, CCE and CPE. Finally, it will be important to launch calls of substantial size to both alleviate gaps in the cancer research continuum and engage exceptional individual PIs, similarly to other funding regiments of proven value, such as Grand Challenges.

## 5 Digitalization

While Gago Conference attendees discussed the status and future of cancer research infrastructures in Europe, as well as all requirements for scaling up existing structures to ensure both innovation and critical mass in terms of data and personnel, digitalization emerged as a key accelerator of developments. Some important concepts for the successful implementation of digitalization in the cancer research and care landscape were discussed by Alfonso Valencia (Barcelona Super Computing Center, Spain), Fatima Carneiro (University of Porto, Portugal), Mark Lawler (Queen's University Belfast, UK), Lena Maier‐Hein (DKFZ, Germany), and Helena Canhão (Nova Medical School, Portugal).

### 5.1 Towards efficient organization of cancer data deposition, sharing and analysis infrastructures

According to Alfonso Valencia, building virtual cohorts will accelerate data discovery and data accessibility across borders, while also ensuring semantic interoperability. For example, the Genomic Data Infrastructure (GDI) initiative is a commitment of 23 European countries to give cross‐border access to 1 million sequenced genomes by 2022. In addition, ELIXIR, Genome Data Alliance and other consortia, including EOSC4Cancer (see also below), are expected to soon provide the infrastructures required for handling genomics, imaging medical, clinical, environmental and socio‐economic data to facilitate research in a federated model. The ongoing collaboration between EOSC4Cancer and UNCAN.EU will ensure the transposition of the infrastructure developments into the future UNCAN.EU cancer federated research hub (see above).

A key challenge in such efforts is to improve data quality and accessibility while also adapting the analysis systems and platforms required by different professionals. In other words, we must make real‐world biological data approachable despite data heterogeneity, complexity, and increasing noise. Innovative technologies moving towards realistic digital twins, for example through simulations of tumour data, single‐cell analyses, and the integration of artificial intelligence (AI) at all levels, may help overcome this challenge. In addition, the integration of data systems in medical practice will help increase tracking of patient trajectories.

While giant technological steps have been taken and the methods to work on virtual data cohorts is currently available, proper organization of such federated models remains to be implemented. The European Genome‐phenome Archive (EGA) may provide a successful example of federated genomic data deposition: data is provided by research centres and healthcare institutions and is maintained in the respective countries while providing controlled access across borders to the participating European countries.

EOSC4Cancer is a Europe‐wide project launched in September 2022 to accelerate data‐driven cancer research. EOSC4C uses colorectal cancer as a model to prepare the roadmap for European Cancer Data Space (EOSC). EOSC4Cancer aspires to integrate cancer registries, screening programmes, cancer research, and clinical trial data. Research software for epidemiological level analysis, software workflows, and portals for patient‐level clinical research and clinical decision systems will also be developed. The EOSC4Cancer initiative will be implemented through six WPs, each focusing on: (a) building federated databases; (b) community‐led technological standards and metadata to enable reproducibility; (c) research portals for data analysis and clinical decision systems; (d) use cases; (e) specialist training; and (f) community engagement.

All infrastructures and initiatives discussed above provide the framework for a successful organizational model that applies to all infrastructures of the cancer research continuum.

### 5.2 Creating knowledge networks

A blueprint guiding the sharing of cancer data must be co‐developed with the organization of federated infrastructures for cancer research and patient data [29]. Mark Lawler emphasized the importance of promoting a citizen‐focused perspective while shaping data‐ sharing policies. Such policies must articulate “a clear social contract”, noted Prof Lawler, where citizens as data donors are also in charge of decision‐making based on newly acquired knowledge [30].

In parallel, multidisciplinary panels of experts are required during data interpretation efforts, as in the Variant Interpretation for Cancer Consortium (VICC) example. The VICC, a project of the Global Alliance for Genomics and Health (GA4GH), led to the establishment of a meta‐knowledgebase of 12 856 aggregate interpretations, introducing the value of open, interoperable sharing of variant interpretation metadata [31]. VICC combined shared genomic and clinical data from six knowledge bases (the Cancer Genome Interpreter Cancer Biomarkers Database (CGI), Clinical Interpretation of Variants in Cancer (CIViC), Jackson Laboratory Clinical Knowledgebase (JAX‐CKB), MolecularMatch (MMatch), OncoKB and the Precision Medicine Knowledgebase (PMKB)).

Empowering a citizen‐centred data‐driven ecosystem and bringing together people from different disciplines has already produced encouraging results through the virtual DATA‐CAN project of Health Data Research UK (HDRUK) [32]. DATA‐CAN is the UK's Health Data Research Hub for Cancer. Rigorous patient involvement in the oversight of all initiatives within the programme has produced better quality projects according to Prof Lawler, who also acts as DATA‐CAN Scientific Director. DATA‐CAN has accelerated the analysis of data on COVID‐19 and cancer in the UK [33], being the first to highlight the impact of COVID‐19 and national lockdowns on cancer systems and cancer patients.

The impact of COVID‐19 on cancer has been dramatic, as within the last 2 years, the pandemic reversed progress that had been achieved for certain cancers in the past two decades. According to the ECO campaign “Time to Act”, 100 million cancer screenings were not performed in Europe during the pandemic, with 1 million cancer cases remaining undiagnosed [9, 34]. The “Time to Act” data navigator collecting data from the ECO campaign has been put into place in a timely manner, highlighting the concept that if we can collect data on COVID‐19 statistics daily, we must start doing the same with cancer and other diseases. Prof Lawler also highlighted upcoming initiatives, including the European Cancer Pulse (an initiative to capture inequalities in Europe to inform cancer policy) and the Lancet Oncology European Cancer GroundShot Commission, the most comprehensive analysis of cancer research activity in Europe to date [9, 35, 36, 37], both of which were launched at the European Cancer Summit in November 2022 in Brussels.

### 5.3 The digital transformation of pathology

Dr Fatima Carneiro presented the tremendous progress made at the front of digital pathology despite the huge costs of maintaining digital space. The Digital Pathology Association, the European Society for Digital and Integrative Pathology, and the Japanese Society of Digital Pathology have taken key initiatives to illustrate the value of digital pathology and provide digital pathology resources and training worldwide.

While digital pathology evolves further, integrating AI, machine learning, deep learning, and artificial neural network tools, regulations must be put in place for its wide implementation. Challenges to the implementation of digital pathology include the building of appropriate infrastructures. Two European projects towards this aim were mentioned by Dr Carneiro as valuable examples of digital pathology infrastructure implementation. The Catalan Health Institute project DigiPatICS has initiated a digital pathology network across eight Catalan hospitals to integrate digital pathology and AI to improve the quality and reproducibility of diagnoses [38]. IPATIMUP has implemented a new digital pathology workflow that might be easily adopted by any pathology laboratory [39].

Broader implementation of digital pathology workflows can help develop new algorithms for analysing whole‐slide imaging, enabling more quantitative analyses. In addition, it is expected to help facilitate a virtual and digital transformation of education, including patient education. While technologies and infrastructures develop, digital pathology also holds promise if combined with other clinical imaging approaches, including digital endoscopy and radiation oncology.

### 5.4 Considerations for the efficient transition towards digital health

While all discussions above highlight the importance of a fast implementation of cancer data deposition, sharing and analysis infrastructures, knowledge networks and digital pathology workflows, there are several requirements for such a digital transition in cancer research and cancer care, as highlighted by Helena Canhão and Lena Meier‐Hein.

Helena Canhão mentioned that electronic medical records, patient registries, shared data repositories, digital tools for early detection, cancer treatment, monitoring of responses to treatment, digital tools for medical appointments, or communication among professionals need to be tightly regulated. Emerging questions also relate to the increasing number of apps and whether they must be regulated as medical devices, in which case validation studies would be needed prior to their implementation. When discussing such digital transitions, we should not forget to consider the human factor, especially regarding communication of diagnosis, patient support, and confidentiality.

In addition, while digital education and training of medical students and healthcare staff emerge as important interventions, and while the involvement of patient associations corresponds to an increase in online searches, we need to pay attention to the quality of the information available. Democratizing access to information through such developments would undeniably help address inequalities but would only do so if the quality of training is prioritized.

Finally, further work is required to improve clinical trial design, as this can facilitate data analysis and help ensure that appropriate formats of patient‐reported data can be integrated into outcomes research. While some aspects of this are already in place, it must be realized that heterogeneity is a key issue, namely, what is working well in one area may not work well in other aspects of digital transformation.

In parallel, Lena Maier‐Hein cautioned about the fast development of AI tools for cancer research data analysis and their limitations. Some AI‐based publications already appear to post false claims, possibly due to errors in perception and interpretations or a lack of appropriate raw data formats. As such, attention must be paid to raw data bias, and validation studies would be needed. In addition, efforts must be invested on correctly structuring and representing data in terms of metadata. External validation must be made an absolute requirement, and splitting the data of a patient cohort into two parts, one for training (i.e. developing a hypothesis) and one for testing purposes, is unacceptable. New validation strategies that rely on data from separate cohorts will dictate how research data are collected. Importantly, validation may require retrospectively having access to the data source, which would imply that data anonymization might pose a limitation.

## 6 Europe‐wide collaboration: Structures, commitment, and resources

In the last session of the 5^th^ Gago Conference, engaging presentations by Michael Baumann, Tit Albreht, Paolo Casali, and Rui Henrique and deep discussions with all participants facilitated the prioritization of the actions needed. Participants examined how to ensure sustainable national and cross‐border structures, secure commitment from all stakeholders involved and wise utilization of personnel and economic resources.

### 6.1 Building sustainable networks of CCCs within Europe

The COVID‐19 pandemic has highlighted the shortcomings of multi‐centre clinical trial structures in sustainably addressing new pressing questions and delivering quality results and scientific impact, even with smaller patient cohorts. This recent example exemplifies the need for developing sustainable research structures within Europe that can rapidly evolve and adapt to new challenges.

A common theme emerges from all discussions reported above: the focus of the next steps in the implementation of functional networks of CCCs and other centres within Europe should be shifted towards how interconnected infrastructures can be developed and sustainably expanded. This was vividly highlighted by Simon Oberst, who noted that future attempts will require a thorough analysis of the ecosystems and good integration within CCCs. Dr Oberst stressed, mentioning the Cancer Research UK Cambridge Centre's Early Cancer Programme, the importance of linking clinicians and patients with researchers of many different disciplines. The Cambridge Early Cancer Programme was initiated by Prof Rebecca Fitzgerald, who brought together other specialities of the Cambridge research ecosystem to later build a network that has successfully spread out nationally and internationally, has developed into a physical institute in Cambridge, and has already delivered a new tool for the early detection of Barrett's oesophagus [40]. As complemented by Rui Henrique (IPO Porto, Porto, Portugal), during the round table discussions, “trust and personal connections can act as glue: cross‐institutional visits, events, and training will help exclude repetition and overlap”.

According to Dr Oberst, the pressing questions of governance and organization of CCCs should also be addressed. According to the experience of the OECI, governance does not necessarily require one legal entity. Beneath an effective Board, collaborative structures (or programmes) need to link clinicians and researchers. Supported by an investment in programme management, this can significantly increase research output in terms of clinical trials and academic publications [41].

A third important issue for ensuring sustainability is for funding bodies, policymakers and governance bodies to always question the value and purpose of introducing a new infrastructure or node. Building a framework will not suffice, sustainability will result from careful and detailed planning involving the above‐mentioned research ecosystem.

### 6.2 Structuring cancer activities in Member States

Michael Baumann, Director of the German Cancer Research Center (DKFZ) and president of EACS, next discussed how the German Cancer Consortium (DKTK) and National Center for Tumor Diseases (NCT) models can serve as successful examples in efforts to structure cancer activities in EU Member States. Historically, Germany features a quite segmented cancer care ecosystem, including private practices, city hospitals, private hospitals, and university hospitals, all complemented by research activities performed at medical faculties and large research institutions such as DKFZ. Across the country, 36 University medical centres provide cancer care in addition to the many other cancer care‐providing hospitals and private practices. The first multidisciplinary CCCs were launched in 2003. A structured competitive programme run since 2007 has helped to increase the number of CCCs officially accredited by Deutsche Krebshilfe to 15, thereby providing nationwide coverage (with several of the CCCs including more than one university medical centre). In 2012, DKTK was introduced as an initiative of the Federal Ministry of Education and Research (BMBF) and linked the DKFZ with 11 University medical centres, and with more than 20 academic and clinical research institutes. These developments aimed to accelerate the transfer of new basic scientific findings towards clinical application and establish a robust national network for cancer research. This approach has also been successfully applied to building networks targeting other diseases, such as neurodegenerative diseases, or diabetes. In such a landscape of increasingly loaded preclinical pipelines, developing of robust structures for early‐phase clinical trials (clinical translational research) has emerged as the key bottleneck in cancer research. NCT was, thus, founded by the DKFZ with partnering university medical centres with the mission to perform research in that bottleneck and involve patients as cancer research partners. NCT features two pilot sites, in Heidelberg and Dresden, and will be expanded to six sites in the coming years while prioritizing synergies, comprehensiveness, critical mass, and overarching investigator‐initiated trials. What has emerged is a strategic partnership between the DKFZ and Deutsche Krebshilfe with a focus on cancer prevention research, a pioneering facility for prevention being in development. This new cancer prevention centre is envisaged to include an outpatient prevention clinic, dedicated laboratories, a training centre, and structures addressing national outreach.

The unique features of the German cancer research landscape that have contributed to the successful implementation of sustainable national networks include cooperative, long‐term institutional funding, the early development of translational hubs, the introduction of a joint funding programme, the establishment of structures for training medical and clinician scientists, and the extended interface of cancer research and cancer care. Importantly, Dr Baumann echoed that setting up a legal entity to govern such a complex infrastructure is not required.

### 6.3 The big picture

The 5^th^ Gago Conference has accomplished the tremendous tasks of summarizing the current EU framework and policy landscape and highlighting next priorities, as perceived by key stakeholders within the European cancer research landscape. While a unified cancer research and cancer care domain is still a work in progress, the closing session of the meeting has helped to spell out open questions while also highlighting priorities and next steps.

According to Tit Albreht, Professor at the National Institute of Public Health, Slovenia and Scientific Coordinator of CRANE, the current understanding is that EBCP will focus on establishing a European Network of CCCs. At the same time, MoC introduced the concept of Comprehensive Cancer Infrastructures (CCIs). Both initiatives present clear recommendations and received strong support from all relevant stakeholders. However, some clear definitions need to be added to their official outlines. What is, for example, the detailed definition and remit of the European Network of CCCs, and how is this different to OECI? The integration of European cancer research centres has progressed through two different paths: one involving a network of CCCs, and one involving a myriad of other organizational forms of networks. Indicatively, the previous Joint Action CanCon (cancercontrol.eu) was defined with the active involvement of OECI, the Comprehensive Cancer Care Network (CCCN), as a network organized around a CCC. In addition, the EC launched several projects that involve networking (JARC, CRANE, JANE, UNCAN, or CCI4EU). Clearly, a mutually agreed definition of a comprehensive cancer control network (under cancer control) is urgently required.

While care comprehensiveness can be ensured via a single CCC structure or through CCC networks, it remains unclear how comprehensive cancer infrastructures are defined. Moreover, it remains to be discussed what a “national” CCC means, and what would be the representation of countries with more than one CCC in a European network. Joint Action CRANE will try to address these issues and propose an organizational and contextual framework for a European network of CCCs.

In detail, CRANE WP5 will focus specifically on the composition, governance, joining process and function of the European Network of CCCs, while CRANE WP6 will approach the organization of high‐quality comprehensive cancer care in networks. WP7 will provide standards and criteria to enable the CCC network. Finally, CRANE WP8 aims to map governance and network solutions for integrated care.

Encouragingly, Dr Albreht emphasized that the focus of both EBCP and MoC on implementation (including implementation research), which should have been more relevant to previous EU programmes, is an extremely positive development. The assessment of feasibility for implementation in every European country and concerted efforts to reduce inequalities will now be key. Importantly, the coordination of the cancer research and cancer policy communities through a network of national hubs is presently missing and arises as the next big priority in the current landscape. The latter will be the focus of a forthcoming Horizon Europe project entitled ECHoS.

Some of the above open questions will perhaps be addressed through JANE and its core WPs. Joint Action was launched in November 2022 and has a duration of 2 years. Key goals include the preparation of a framework for the new Networks of Expertise and the critical evaluation of existing models of European networking. These networks, as also presented in further detail by Paolo Casali, Instituto Nazionale Tumori, Milano, Italy, will each expand expertise on complex or poor prognosis cancer, primary prevention, cancer survivorship, palliative care, omics technologies, hi‐tech medical resources, and cancer in adolescents and young adults, reinforcing CCC networks and the cancer community.

It remains to be defined how JANE will achieve sustainability, integration between healthcare and research, integration between EU networking and member states, integration between digital technologies and electronic health records of the various institutes, as well as patient engagement – all important aspects, where, as Dr Casali noted, previous European reference networks dealing with rare cancers (such as JARC) have failed.

## 7 Conclusions and comments

The Gago conference discussed the ongoing implementation of Europe's Beating Cancer Plan (EBCP) and the Mission on Cancer (MoC). The MoC Board has a broad approach and emphasizes the analyses of the innovation process by Mariana Mazzucato, which is highly dependent on public funding mechanisms. A promising initiative is the UNCAN which will set the agenda for international research collaboration covering the most important research areas. The increased interest in health‐related quality‐of‐life research, including cancer survivorship, is very positive, and the expansion of digitalization and data science offers several new possibilities for prevention, therapeutics and cancer research.

Problems needing additional attention were identified:

- While the economic consequences of the increasing cancer problem are huge, a missing element is the essential outcomes research necessary for health economics analyses to develop cost‐effectiveness. One of the main problems is the drug development process which needs to deliver information on the clinical effectiveness of new anticancer agents.
- With patients at the centre, multidisciplinary cancer treatment and care requires continuous innovation with translational research covering all cancer treatment/care components. Around 35 accredited CCCs in the EU have this mission, but at least 100 are needed. There is no clear strategy for developing CCCs to cover the EU member states and reach all cancer patients. Further, there need to be more definitions of CCC‐related terms, like CC networks and infrastructures. The responsibility of a CCC for an outreach area needs to be clarified if inequalities will be limited.
- International research collaborations aiming to develop personalized/precision cancer medicine have started. However, it is challenging to structure the research, which requires advanced infrastructures for the stratification of patients available only in cutting‐edge cancer research centres while treatment patients should be where they live.
- There is an increased understanding of expanding funding for prevention research, including implementation research. With the effective use of present evidence, a large part of the cancer problem can be eliminated. An important area to develop, which should have been discussed in detail, is the prevention screening strategies. Screening for early detection of colorectal and cervical cancer is effective due to the strong focus on identifying precursor lesions, which hopefully will be more considered for other cancer diagnoses.
- The specific discussion on science policy had a broad and positive character. However, to mitigate the inequality problem in the EU, implementation of present evidence regarding prevention, therapeutics and care should be a task addressed together with the EBCP. The fact that advanced research centres expand innovations in basic biological and technological research with increasing inequalities as consequences needs specific policy actions for structuring the international research collaborations towards personalized/precision cancer medicine.

## Conflict of interest

Where authors are identified as personnel of the International Agency for Research on Cancer/World Health Organization, the authors alone are responsible for the views expressed in this article and they do not necessarily represent the decisions, policy or views of the International Agency for Research on Cancer/World Health Organization. Elena Garralda reports consultant honoraria from Roche/Genentech, F. Hoffmann‐La Roche, Ellipses Pharma, Neomed Therapeutics Inc., Boehringer Ingelheim‐Janssen Global Services, AstraZeneca, SeaGen, TFS‐Alkermes, Thermo Fisher, Brristol‐Mayers Squibb, MabDDiscovery, Anaveon, F‐Star Therapeutics, Hengrui, Sanofi; research funding from Novartis/Roche, Thermo Fisher, AstraZeneca, Taihho, BeiGene; is a principal investigator/subinvestigator of clinical trials for Adaptimmune LLC, Affimed Gmbh, Amgen SA, Anaveon AG, AstraZeneca AB, Bicycletx Ltd, BioInvent International AB, Biontech SE, Biontech Small Molecules Gmbh, Boehringer Ingelhem International Gmbh, Catalym Gmbh, Cyclacel Biopharmaceuticals, Cytovation AS, Cytomx, Dot Therapeutics, F.Hoffmann La Roche Ltd, F‐Star Beta Limited, Genentech Inc, Genmab B.V., Hifibio Therapeutics, Hutchison Medipharma Limited, Icon, Imcheck Therapeutics, Immunocore Ltd, Incyte Corporation, Incyte Europe Sàrl, Janssen‐Cilag International NV, Janssen‐Cilag SA, Laboratorios Servier SL, Medimmune Llc, Merck & Co, Inc, Merck Kgga, Novartis Farmacéutica, S.A, Peptomyc, Pfizer Slu, Relay Therapeutics, Replimmune, Ribon Therapeutics, Ryvu Therapeutics SA, Seattle Genetics Inc, Sotio as, Sqz Biotechnologies, Symphogen A/S, Taiho Pharma Usa Inc, T‐Knife Gmbh. Mark Lawler declares honoraria unrelated to this work from Bayer, Carnall Farrar, EMD Serono, Novartis, Pfizer and Roche.
